# Applications of Genome Editing Technology in Research on Chromosome Aneuploidy Disorders

**DOI:** 10.3390/cells9010239

**Published:** 2020-01-17

**Authors:** Silvia Natsuko Akutsu, Kazumasa Fujita, Keita Tomioka, Tatsuo Miyamoto, Shinya Matsuura

**Affiliations:** Department of Genetics and Cell Biology, Research Institute for Radiation Biology and Medicine, Hiroshima University, Hiroshima 734-8553, Japan; silvia-akutsu@hiroshima-u.ac.jp (S.N.A.); kzfujita@hiroshima-u.ac.jp (K.F.); d173695@hiroshima-u.ac.jp (K.T.)

**Keywords:** chromosome aneuploidy disorder, genome editing, chromosome elimination, iPSC reprogramming

## Abstract

Chromosomal segregation errors in germ cells and early embryonic development underlie aneuploidies, which are numerical chromosomal abnormalities causing fetal absorption, developmental anomalies, and carcinogenesis. It has been considered that human aneuploidy disorders cannot be resolved by radical treatment. However, recent studies have demonstrated that aneuploidies can be rescued to a normal diploid state using genetic engineering in cultured cells. Here, we summarize a series of studies mainly applying genome editing to eliminate an extra copy of human chromosome 21, the cause of the most common constitutional aneuploidy disorder Down syndrome. We also present findings on induced pluripotent stem cell reprogramming, which has been shown to be one of the most promising technologies for converting aneuploidies into normal diploidy without the risk of genetic alterations such as genome editing-mediated off-target effects.

## 1. Introduction

### 1.1. Human Genetic Aneuploidy Disorders

Eukaryotic cells have developed a surveillance mechanism to ensure accurate chromosomal segregation during cell division. Errors during meiosis or mitosis can generate daughter cells with an abnormal number of chromosomes, a phenomenon called “aneuploidy” (i.e., not euploidy). Aneuploid cells exhibit gain or loss of a whole chromosome, causing abnormalities in embryonic development and a predisposition to cancer [1,2,3].

The risk of embryonic aneuploidies leading to miscarriage or congenital disorders generally increases with maternal age [4,5,6,7]. The overall incidence of chromosomal anomalies in neonates is approximately one out of 160 births [8,9,10,11,12]. Monosomies (possessing only one chromosome of a pair) are basically deleterious due to the level of gene expression being insufficient for cell survival; most such cases, except for monosomy X, are thus embryonically lethal [13]. In contrast, live births occur for trisomies of chromosomes 13, 18, 21, X, and Y, owing to the smaller number of genes encoding proteins located on these chromosomes in comparison to the other autosomal chromosomes [14,15,16,17,18]. However, trisomy 13 and trisomy 18 have severe phenotypic consequences, which are rarely compatible with long-term survival [19,20,21,22]. Here, we address genome editing technology as a potential therapy for aneuploidy disorders.

### 1.2. Autosomal Chromosomes

#### 1.2.1. Trisomy 21 (Down Syndrome)

Down syndrome (DS) is the most common trisomy disorder among live-born infants with aneuploidy. DS patients show a characteristic facial appearance, intrauterine growth restriction (IUGR), intellectual disability, and an increased risk of leukemia [23,24,25,26,27]. In general, the specific set of genes causing the clinical phenotypes in trisomy syndromes affected by the increase in chromosome copy number is largely unknown. However, it has been reported that three copies of the *RUNX1*, *ETS2*, and *ERG* genes on chromosome 21 interplay with somatic *GATA1* mutations on the X chromosome to increase the risk of leukemia in DS. Interestingly, TALEN-mediated elimination of the *GATA1* mutation in DS patient-derived induced pluripotent stem cells (iPSCs)restored the proper hematopoiesis even in the presence of trisomy 21 [28].

#### 1.2.2. Trisomy 18 (Edwards Syndrome)

Trisomy 18 is the second most common trisomy among live-born infants with aneuploidy. Its features include IUGR, hypertonia, prominent occipital bone, small mouth, micrognathia, short sternal bone, horseshoe kidney, small pelvis, and clenched fists with second and fifth fingers overlapping [29,30,31].

#### 1.2.3. Trisomy 13 (Patau Syndrome)

Trisomy 13 patients show clinical features due to an early defect in the development of the prechordal mesoderm, leading to midline malformations including holoprosencephaly, absence of the olfactory nerve and bulb, severe eye defects, deafness, and midline cleft lip and palate. In addition, IUGR, omphalocele, genitourinary anomalies, hemangiomas, and polydactyly often appear in these patients [32,33,34,35].

### 1.3. Sex Chromosomes

#### 1.3.1. Turner Syndrome (45,X)

Although the autosomal monosomies are lethal, monosomy of the X chromosome underlying Turner syndrome can be associated with live birth and viability despite a high rate of fetal loss. This syndrome shows a type of hypergonadotropic hypogonadism characterized by a female phenotype, gonadal dysgenesis, and sexual immaturity [36]. Clinical features include intense intrauterine edema with nuchal edema, growth retardation, short stature, broad chest, no development of secondary sexual characteristics in adulthood, facial appearance of epicanthic folds, ocular hypertelorism, thick eyebrows, low implanted ears, micrognathia, low posterior hairline, and renal malformations [37,38,39].

#### 1.3.2. Klinefelter Syndrome

Klinefelter syndrome is one of the most common forms of hypergonadotropic hypogonadism in men. It is due to the presence of one extra X chromosome (47,XXY) or, more rarely, two or three extra X chromosomes (48,XXXY or 49,XXXXY). Secondary sexual characteristics are poorly developed, and patients show small testicles associated with azoospermia/oligospermia, gynecomastia, and infertility because of lower levels of testosterone and hypogonadism [36,40]. Patients also exhibit a tall and thin stature, with long limbs and reduced muscle mass [41,42,43].

There are other aneuploidies (cited in Table 1) that are even rarer than those previously mentioned and have been described in some case reports in the literature [31,44,45,46,47,48]. Most of the other autosomal aneuploidies exhibit mosaicism, making them compatible with life, since full trisomy is lethal. The main clinical features of aneuploidy disorders include impairments of cognitive development and growth [2,49,50]. More than 30% of individuals with numerical chromosomal abnormalities have cardiac malformations [51,52,53,54].

### 1.4. Mosaic Variegated Aneuploidy (MVA)

The constitutional aneuploidy disorders described in Section 1.1. and Section 1.2. are pathologically based on stochastic chromosome mis-segregations in gametogenesis and early embryogenesis. In contrast, in patients carrying mutations (Mendelian inheritance) in the genes underlying proper chromosome segregation, the entire correction mechanism is compromised, generating a pool of cells with mosaic variegated aneuploidy (MVA) [55,56]. MVA is a rare autosomal recessive disorder that presents different sets of aneuploid somatic cells due to germline mutations in genes important for the surveillance of chromosomal segregation [57,58,59]. To date, MVA has been categorized into three types according to the causative genes.

#### 1.4.1. MVA1 or MVA1 Syndrome

Premature chromatid separation (PCS)/MVA1 syndrome is caused by germline mutations in the *BUB1B (15q15.1)* gene [60,61,62]. This gene encodes BubR1 protein, a central player in the mitotic spindle assembly checkpoint (SAC). Insufficiency of BubR1 protein causes impaired SAC, chromosome alignment defects, and chromosome segregation errors characterized by the premature chromatid separation of all chromosomes and MVA generating monosomies, trisomies, and/or double trisomies for multiple different chromosomes. Patients are characterized by IUGR, microcephaly, uncontrollable seizures, mental retardation, Dandy–Walker anomaly, early onset of cataracts, polycystic kidney, and a strong predisposition for childhood cancers (Wilms tumor, rhabdomyosarcoma, or leukemia) [63,64,65,66,67,68,69].

#### 1.4.2. MVA2

The *CEP57(11q21)* gene encoding a centrosomal 57 kDa protein required for microtubule attachment to centrosomes, chromosome misalignment, and multipolar spindles is the causative gene of MVA2 [70,71,72]. The clinical features of patients with MVA2 are IUGR, microcephaly, developmental delay, and mild rhizomelic shortening of the upper limbs. A predisposition for cancer has not been detected in MVA2 patients [73,74,75].

#### 1.4.3. MVA3

*TRIP13(5p15.33)* encodes a protein involved in SAC, thyroid hormone receptor interactor 13, which works through inactivation of the MAD2 protein. Germline mutations of the *TRIP13* gene cause MVA3 [76]. Cells from patients show a high frequency of premature chromatid separation, like in MVA1 patients. The clinical features are IUGR, microcephaly, developmental delay, mild dysmorphism, seizures, abnormal skin pigmentation, and a predisposition for Wilms tumor and leukemia [77,78].

## 2. Gene Targeting-Mediated Chromosome Elimination

The aneuploidy disorders have been considered to be irremediable. However, recent studies using genetic engineering have revealed the possibility of performing aneuploidy therapy in cultured cells. Here, we summarize some of the main studies aimed at eliminating an entire chromosome using the Cre/loxP system [79,80,81,82,83,84,85], the *TKneo* transgene for positive and negative antibiotic selection [86], CRISPR/Cas9 system-mediated multiple cleavage [87,88], and zinc finger nuclease (ZFN)-mediated knock-in of the *XIST* gene to silence one copy of chromosome 21 [89] (cited in Table 2).

### 2.1. Cre/loxP System-Mediated Chromosome Elimination

**Figure 1:**
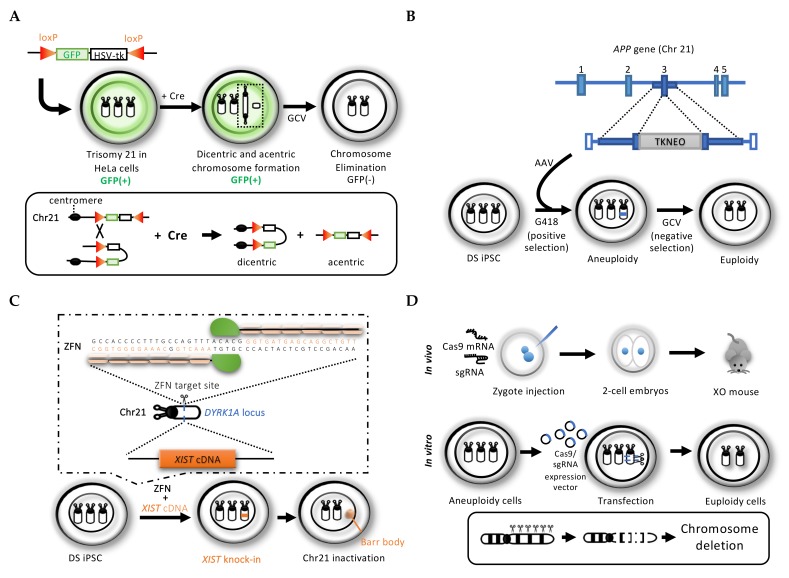
Schematic overview of elimination of extra chromosome 21 (chr 21) using genome editing technology. (**A**) Integration of the *GFP (EGFP)* and *HSV-tk* gene cassette surrounded by two inverted loxP sites on the homologous arms of chr 21: Cre-dependent recombination between the sister chromatids with inverted loxP generates unstable dicentric and acentric chromosomes for chromosome elimination. (**B**) Knock-in of the *TKneo* gene cassette into the *amyloid precursor protein (APP)* gene exon 3 target locus of one extra copy of chr 21 in Down syndrome (DS)-iPSCs enabled the correction of aneuploidy, followed by positive drug and negative using G418 (neomycin) and GCV (ganciclovir), respectively. (**C**) Zinc Finger Nuclease (ZFN)-mediated *XIST* gene knock-in on the *Dual specificity tyrosine phosphorylation regulated kinase 1A (DYRK1A)* gene locus of chr 21 induced Barr body formation to silence the extra copy of chr 21 in DS-iPSCs. (**D**) CRISPR/Cas9 system targeting the unique repeat sequences introduces multiple DNA double-strand breaks (DSBs) into the target chromosome for deletion of the entire chromosome; XY mouse zygotes injected with Cas9 mRNA and sgRNA to the repeat sequence on the X chromosome for the generation of XO mice in vivo; and DS iPSCs transfected with CRISPR/Cas9 expression vector for multiple cleavages into the extra copy of chr 21 in vitro.

A single loxP site contains two 13-bp inverted repeats flanking an asymmetric 8-bp core sequence recognized by Cre recombinase [90]. Integration of the inverted loxP into the chromosomes were performed by conventional gene targeting, CRISPR/Cas9 nickase system, and TALEN technology, respectively [82,83,91]. Chromosomes with inverted loxP sites can be converted to unstable dicentric and acentric chromosomes in a Cre recombinase-dependent manner (Figure 1). These unstable chromosomes will be excluded during cell division, thereby promoting elimination of the target chromosome with the inverted loxP sites (Figure 1A). Lewandoski et al. [83] first used this strategy to demonstrate the elimination of the Y chromosome in XY male mice in vivo. Male mice bearing a Y chromosome with inverted loxP sites were mated with female transgenic Cre mice to eliminate the Y chromosome during embryogenesis, thereby developing as XO female mice. Moreover, Matsumura et al. [85] applied a chromosome elimination cassette (CEC), featuring fluorescent-protein and puromycin resistance genes surrounded by inverted loxP sites into chromosome deletions in cultured cells in vitro. Mouse tetraploid (4*n* = 80,XXXY) ES cells fused with two diploid ES cells differentiated but did not proliferate [79,85]. The elimination of two copies of chromosome 6 tagged with CEC in the tetraploid ES cells enabled survival in an undifferentiated state and the capability of teratoma formation, implying that chromosome 6 including the *Nanog* gene is indispensable for the self-renewal and pluripotency of mouse ES cells.

To dissect the molecular pathology of chromosome 21-associated gene-dosage imbalance in Down syndrome, Sato et al. [82] used CEC cassette-tagged trisomy 21 HeLa cells to generate normal disomic cells (Figure 1A). A cassette of *green fluorescent protein (GFP)* and *herpes simplex virus-thymidine kinase* (*HSV-tk*) genes surrounded by inverted loxP sites was integrated into one copy of chromosome 21 in the trisomy HeLa cells by homologous recombination following CRISPR/Cas9 system-mediated DNA nicking. The CEC-tagged trisomy HeLa cell clones were positively selected by the GFP signal. Cre recombinase induced sister-chromatid recombination in the inverted loxP site-tagged chromosome to generate unstable dicentric and acentric chromosome 21. The disomy 21 HeLa cells in which the extra copy of chromosome 21 had been eliminated were negatively selected by the antiviral drug ganciclovir (GCV), which kills cells in the presence of thymidine kinase.

### 2.2. Conventional Gene-Targeting Mediated Dual Drug Selection Cassette Knock-In

Introduction of a gene cassette for positive and negative drug selection into one copy of chromosome 21 via a conventional gene-targeting method demonstrated the correction of trisomy to disomy in Down syndrome (DS) patient-derived induced pluripotent stem cells (iPSCs). Li et al. [86] introduced the *thymidine kinase and neomycin resistant* (*TKneo*) transgene into one copy of the *amyloid precursor protein (APP)* gene exon 3 target locus on chromosome 21 in DS-iPSCs using adenovirus vector (AVV). The DS iPSC clones with the cassette were positively selected by G418 (neomycin) treatment at a rate of 0.14%. The disomy 21 DS-iPSC clones were negatively selected by GCV at a rate of ~10^−4^, thereby effectively generating normal disomic cells (Figure 1B). Trisomy 21 is known to disturb the endothelial differentiation to cause impaired angiogenesis in DS [92]. In the teratoma formation assay, the disomy 21 DS-iPSCs developed endothelial tubes normally [86].

### 2.3. Genome Editing Technology for Rescuing Trisomy In Vitro and In Vivo

#### 2.3.1. Zinc Finger Nuclease (ZFN) Mediated the XIST Gene Knock-In to Silence an Extra Chromosome 21 in Down Syndrome Patient Cells

The *XIST* gene located on the human X chromosome exclusively inactivates one copy of the X chromosome in mammalian females (XX) as a natural mechanism to adjust the gene dosage between females and males [93]. The X chromosome inactivation is cytologically manifested as a condensed Barr body. In addition to the elimination of extra chromosomes, Jiang et al. [89] reported another potential chromosomal therapy for Down syndrome using a ZFN-mediated *XIST* gene knock-in technology. The ZFNs enabled the accurate and efficient insertion of the *XIST* gene into chromosome 21 in DS iPSCs. The rates of successful single, double, and triple knock-in of the *XIST* gene into the target locus in the DS-iPSCs were 87.7%, 10.8%, and 0.0%, respectively. The doxycycline-inducible full-length *XIST* transgene was introduced into the *DYRK1A* intron 1 locus on chromosome 21q22. The ZFN-mediated *XIST* transgene integration into the *DYRK1A* locus in two and three alleles occurred effectively, while the transgene insertion into only one allele was rare. Further studies are needed to develop genome editing technology for controlling the copy number of transgene insertion. In contrast, the doxycycline control component (rtTA) transgene was inserted into the *AAVS1* safe harbor site on chromosome 19. The doxycycline-dependent *XIST* gene expression induced “chromosome 21 Barr body” to inactivate the extra chromosome 21 in DS-iPSCs (Figure 1C). Gene expression in the DS iPSCs was identical to that in the normal individual iPSCs. However, several genes on chromosome 21 escaped from the *XIST*-mediated inactivation to maintain the overdose transcript amounts of aneuploid cells. Recently, it was reported that trisomy 21 upregulates insulin-like growth factor (IGF) signaling to enhance the *GATA1* expression aberrantly, thereby causing leukemia in DS. The *XIST*-mediated trisomy 21 silencing in DS patient-derived iPSCs restored the proper hematopoietic differentiation in vitro [94]. For clinical applications of this method, further improvements are required for the complete silencing of targeted chromosome 21.

#### 2.3.2. CRISPR/Cas9 System to Introduce Multiple DNA Cleavages for Target Chromosome Elimination

To eliminate extra chromosomes, multiple guide RNAs of the CRISPR/Cas9 system for introducing DNA double-strand breaks (DSBs) into the target site are designed for an entire region of the target chromosome [95,96,97,98,99]. Adikusuma et al. [88] used this strategy to delete the mouse Y chromosome in vitro and in vivo (Figure 1D). Guide RNAs corresponding to repetitive sequences (from 40 to 140 tandem repeats) of the centromere and long arm of chromosome Y were used in their study. Zuo et al. [87] also designed two other sgRNAs in repetitive elements of the *Rbmy1a1* and *Ssty2* genes of the mouse Y chromosome to generate XO mice and embryonic stem cells (ESCs) as Turner syndrome models with approximately 20% efficacy. In mESCs with human chromosome 14 (hChr14) established by the artificial chromosome transfer method, six sgRNAs targeting repetitive elements on the long arm of hChr14 were sufficient to achieve CRISPR/Cas9 system-mediated hCh14 elimination. The elimination frequencies were around 10%. Interestingly, this strategy was applied to human cancer cell line HT-29 with four copies of chromosome 7 (hChr7 = 4) to reduce the copy number and to thereby inhibit malignant proliferation. Notably, it was reported that CRISPR/Cas9 system-mediated chromosomal elimination using two sgRNAs targeting repetitive sequences on chromosome 21 in the DS-iPSCs converted trisomy 21 to disomy at a rate of approximately 15% (Figure 1D). However, the off-target effects of the CRISPR/Cas9 system and the efficacy of aneuploidy rescue used in this approach should be evaluated and improved for application to basic and clinical research on aneuploidy disorders.

## 3. Reprogramming-Mediated Karyotype Correction

The genetic manipulations of aneuploidy described above have provided new insights for overcoming various diseases. However, these approaches must be accompanied by a risk of off-target alterations to the genome. Recently, as an alternative approach that avoids these potential problems, it has been reported that iPSC reprogramming potentially corrects structural and numerical chromosomal anomalies [100,101,102].

### 3.1. Cell-Autonomous Correction of Ring Chromosome During iPSC Reprogramming

**Figure 2:**
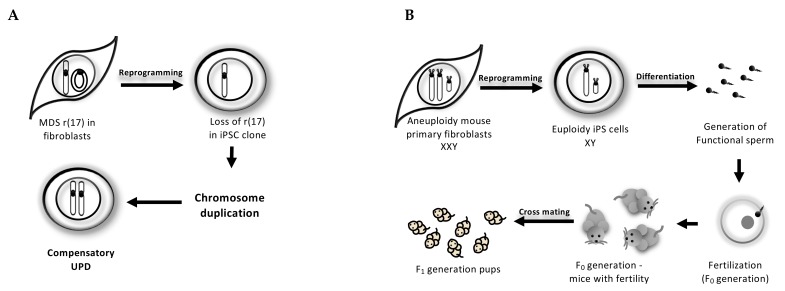
Schematic overview of iPSC reprogramming-mediated chromosome correction. (**A**) Cell-autonomous correction of the ring chromosome 17 (r(17)) in Miller–Dieker syndrome (MDS) through the loss of abnormal ring chromosome and compensatory uniparental disomy (UPD) mechanism during the reprogramming process. (**B**) iPSC reprogramming-mediated trisomy-biased chromosome loss corrects the XXY aneuploidy mouse primary fibroblasts from Klinefelter syndrome model mice to rescue the infertility. Euploid XY iPSCs are differentiated into the functional sperms capable to generate the F1 and F2 generation-pups.

Miller–Dieker syndrome (MDS) is caused by heterozygous deletion of human band ch17p13.3, and some MDS patients carry ring chromosome 17 with the 17p13.3 deletion [103]. MDS patients have several clinical phenotypes, including brain malformation, and mental and growth retardation [104]. When primary fibroblasts from an MDS patient with ring chromosome 17 were reprogrammed into iPSCs, the ring chromosome 17 was lost and, instead, another chromosome 17 was duplicated in four out of six clones, thereby establishing the uniparental disomy (UPD) of chromosome 17 (Figure 2A). In this approach of ring chromosome correction, the set of chromosomes is numerically corrected while the expression of the imprinted genes on the UPD chromosomes might not be rescued. Therefore, it is necessary to develop other UPD-independent approaches for ring chromosome correction [101,105,106] (Figure 2A).

### 3.2. Trisomy-Biased Chromosome Loss (TCL) to Convert the Trisomy into Disomy During iPSC Reprogramming

Hirota et al. [102] demonstrated that aneuploidy was corrected at a rate of approximately 20% during the process of reprogramming Klinefelter syndrome-modeled XXY mouse primary fibroblasts into iPSCs. The euploid XY iPSCs generated from Klinefelter syndrome model mice differentiated into functional testicular sperm (Figure 2B). Notably, the trisomy-biased chromosome loss through iPSC reprogramming was also enforced in DS iPS cells, which lost the extra copy of chromosome 21. Interestingly, the long-term passage of DS-iPSCs with trisomy 21 also led to TCL, thereby correcting the karyotype. However, the mechanisms that causes TCL remains unclear.

## 4. Concluding Remarks

In this review, we have provided an overview of the deletion or silencing of an extra chromosome copy using genome editing technology, cell-autonomous correction of ring chromosomes, and TCLs during the reprogramming process in constitutional aneuploidy disorders. However, most of these approaches still have low efficacy [94,105].

There is still a gap between genome editing technology-mediated trisomy rescue in vitro and its clinical applications. Since it is too difficult to evaluate the on-target efficacy and risk of off-target effect by genome editing in vivo and to deliver the genome editing tools into the target tissues, the safety of genome editing therapy in human bodies should be improved. That is why it is not practical to apply chromosome correction methods in cultured cells into aneuploidy disorder patients. In contrast, ex vivo genome editing therapy has been advancing because the quality of genome-edited cells can be checked before introducing them into the patients. For examples, genome editing technology in the hematopoietic cells such as T cells ex vivo have been used to overcome HIV infection [107] and leukemia progression [108]. In the future, applications of genome editing technology-mediated trisomy rescue mentioned in this review into T cells from Down syndrome patients might contribute to reducing or overcoming the risk of leukemia.

Since MVAs have different random and multiple aneuploidies, it is not practical to apply the genome editing technology-mediated chromosome correction mentioned in this review. Importantly, the iPSC reprogramming of mouse embryonic fibroblasts (MEFs) from *BubR1* hypomorphic mice did not correct MVA [109], implying that BubR1 might be required for maintenance of the karyotype after chromosome number rescue accompanied with iPSC reprogramming. For the potential therapy for MVA1 patients, it might be useful to correct the *BubR1* mutations using genome editing technology.

In conclusion, we addressed several approaches that may contribute to future therapy for aneuploidy disorders. Further studies are needed to improve the efficacy of aneuploidy correction using genome editing technology and iPSC reprogramming for the clinical applications of these approaches.

## Figures and Tables

**Table 1:** Human Chromosome Aneuploidy Disorders.

Disease	Inheritance	Causative Gene (Gene Ontology)	Chromosome Imbalance	Frequency of Patients	Clinical Features				
					Congenital Heart Defect	Microcephaly	Mental Retardation	Cancer Predisposition	Others
**Autosomal chromosome**									
Down syndrome	IC		T 21	1/750 live births	+	−	+	+	upward-slanting palpebral fissures, epicanthal folds, single palm fold
Edwards syndrome	IC		T 18	1/6000–1/8000 live births	+	+	unknown	unknown	prominent occiput, low-set malformed ear, micrognathia
Patau syndrome	IC		T 13	1/20,000 live births	+	+	unknown	unknown	polydactyl, midline cleft lip, flexion of the fingers, polycystic kidneys
Mosaic trisomy 8	IC		Mosaic T 8	>100 cases reported	+	−	+	+	morphological brain abnormalities, high arched or cleft palate, micrognathia, renal malformation
Mosaic trisomy 9	IC		Mosaic T 9	>40 cases reported	+	−	+	+	morphological brain abnormalities, micrognathia, Dandy–Walker malformation, renal malformation
Mosaic trisomy 22	IC		Mosaic T 22	>20 cases reported	+	+	+	unknown	hemi dystrophy, midfacial hypoplasia, cleft palate, micrognathia, renal hypoplasia
**Sex chromosome**									
Turner syndrome	IC		M X	1/2000–1/5000 live female births	+	+	+	−	posteriorly rotated ears, neck webbing, broad chest, short stature, micrognathia
Klinefelter syndrome	IC		add chr X in male	1/426–1/1000 live male births	−	−	+	−	tall stature, long limbs, hypogonadism, infertility
XXX syndrome	IC		T X	1/900 live female births	−	−	−	−	tall stature, normal fertility
XYY syndrome	IC		add chr Y in male	1/800–1/1000 live male births	−	−	−	−	tall stature, hyperactive behavior, distractibility, temper tantrums, low frustration tolerance
**Mosaic Variegated Aneuploidy (MVA)—autosomal and sex chromosome**									
MVA1 or MVA	AR	*BUB1B* (mitotic SAC)	M, T, and double T	<1/1,000,000 live births	+	+	+	+	Dandy–Walker complex, cataracts, premature aging, multiple renal cysts
MVA2	AR	*Cep57* (spindle pole integrity)	M, T, and double T	5 cases reported	+	+	+	−	rhizomelic shortening of the upper limbs, skull anomalies
MVA3	AR	*TRIP13* (mitotic SAC)	M, T, and double T	6 cases reported	−	−	+	+	seizures, abnormal skin pigmentation, arthrogryposis

IC: isolated cases; AR: autosomal recessive; T: trisomy; M: monosomy; add: additional; SAC: Spindle Assembly Checkpoint.

**Table 2:** Gene targeting-mediated chromosome elimination and genome editing technology.

Used Genome Editing System	Aneuploidy Focused	Purpose	Cell Type	Target Gene Locus	Transgene	Selection Method	Initial → Final Genotype	Reference
**Cre/inverted loxP**	XY genotype	Chr del	Mouse zygotes	chr Y	Y-inverted loxP transgene	−	XY → XO	[83]
	Tetraploid mESC	Chr del	mES somatic hybrid cells	chr 11, chr 12, chr 6	CEC	Puro drug selection, sorting by FACS	40,XY (2*n*) → 80,XXYY (4*n*)→ 79,XXYY (4*n*)	[85]
	Tetraploid mESC	Chr del	Hybrid cells from two CEC transgenic ESC lines (CEC-ESC)	chr 6, chr 11, chr 12, chr 17	CEC	Puro and neo drug selection, sorting by FACS	80,XXXY (4*n*) → 78,XXYY (4*n*)	[84]
	CEC-mESC	Chr del	Transgenic mESC containing a copy of CEC (CEC-ESC)	chr 5 (band F), chr 13 (band A)	CEC	Sorting by FACS	40,XY → 39,XY	[80]
	Down syndrome	Chr del	HeLa cells with three copies of chr 21	intergenic region between *RCAN1* and *CLIC6* genes	loxP-*HSV-tk*	GCV drug selection	47,+21 → 46	[82]
	Tetraploid MEF	Chr del	Tetraploid immortalized murine embryonic fibroblasts	chr 9, chr 10, chr 12, chr 14	GFP-inverted loxP-*hDC2*	Sorting by FACS	40,XY (2*n*) → 80,XXYY (4*n*) → 79,XXYY (4*n*)	[79]
**Conventional gene targeting**	Down syndrome	Knock-in	Down syndrome hiPSC	*APP* gene	*TKNEO*	Neo and GCV drug selection	47,+21 → 46	[86]
**ZFNs**	Down syndrome	Silencing the chr 21	Down syndrome hiPSC	*DYRK1A* gene	*XIST*	Puro drug selection	47,+21 → 47,+21(chr Barr)	[89]
**CRISPR/Cas9**	XY genotype	Chr del	mESCs	SRE of centromere and long arm of chr Y	−	Puro drug selection	XY → XO	[88]
	XY genotype	Chr del	mESCs	SRE of *Rbmy1a1* and *Ssty2* genes	−	Sorting by FACS	XY → XO	[87]
	XY genotype	Chr del	Mouse brain	SRE of *Rbmy1a1* and *Ssty2* genes	−	Sorting by FACS	XY → XO	[87]
	Turner syndrome	Chr del	Mouse zygotes	SRE of *Rbmy1a1*, *Ssty1*, and *Ssty2* genes	−	−	XY → XO	[87]
	Turner syndrome	Chr del	Mouse zygotes	SRE of long arm of chr X	−	−	XX → XO	[87]
	mESC aneuploidy	Chr del	Stable mESC line with an extra human chr 14 established by chr transfer	SRE of long arm of chr 14	−	Sorting by FACS	mChr14 = 1 → mChr14 = 0	[87]
	Down syndrome	Chr del	mESCs with trisomy 21/hiPSCs with trisomy 21	SRE of long arm of chr 21	−	Sorting by FACS	47,+21 → 46	[87]
	Cancer	Chr del	Human cancer cell line HT-29	SRE of short and long arm of chr 7	−	Sorting by FACS	hChr7 = 4 →hChr7 = 3	[87]

Chr: Chromosome; del: deletion; mESCs: mouse Embryonic Stem Cells; CEC: Chromosome Elimination Cassette; hiPSC: human iPSC; MEF: Mouse Embryonic Fibroblasts; SRE: Sequences in repetitive elements; FACS: Fluorescence-activated cell sorting.
